# Intralesional ALA-PDT for residual extramammary Paget's disease: a case report

**DOI:** 10.3389/fsurg.2026.1828223

**Published:** 2026-06-30

**Authors:** Jianhua Huang, Dongya Wang, Yiwu Yu, Szeman Cheung, Li Xiao, Fei Miao, Wenjing Zha, Yitao Qian, Ke Li, Lei Shi

**Affiliations:** 1 Department of Dermatology, Huadong Hospital, Fudan University, Shanghai, China; 2 Department of Urology, Huadong Hospital, Fudan University, Shanghai, China; 3 Department of Pathology, Huadong Hospital, Fudan University, Shanghai, China

**Keywords:** 5-aminolevulinic acid, drug delivery, extramammary Paget's disease, intralesional photodynamic therapy, residual disease

## Abstract

**Background:**

Extramammary Paget's disease (EMPD) is a rare cutaneous malignancy often associated with positive surgical margins and high local recurrence rates. While wide local excision remains the standard treatment, re-excision may be impractical in anatomically sensitive areas. Topical photodynamic therapy (PDT) is frequently used for superficial lesions but its efficacy is limited by poor photosensitizer penetration, particularly when tumor cells invade deep into the dermis or adnexal structures.

**Case presentation:**

An elderly male with scrotal EMPD underwent surgical excision, but postoperative pathology revealed positive margins with tumor infiltration into the subcutaneous fat. Further wide excision was deemed unfeasible due to functional and anatomical constraints. Pre-treatment high-frequency ultrasound demonstrated a lesion thickness of approximately 2.7 mm, exceeding the effective penetration depth of topical PDT. To overcome this barrier, intralesional 5-aminolevulinic acid photodynamic therapy (ALA-PDT) was administered monthly. A 10% ALA solution was injected directly into the residual plaque, followed by a 2-hour incubation and irradiation with 630 nm red light at a fluence of 120 J/cm^2^. After three sessions, the indurated erythematous plaque showed marked thinning and fading; complete clinical regression was achieved after six sessions. The treatment was well-tolerated, with only mild pain and transient erythema. No recurrence was observed during the 6-month follow-up period.

**Conclusion:**

Intralesional ALA-PDT is a highly effective, minimally invasive, and tissue-preserving treatment for residual EMPD with deep dermal invasion. By ensuring adequate photosensitizer delivery throughout the entire lesion, it overcomes the penetration limitations of topical PDT and represents a valuable option when surgery is not feasible.

## Introduction

Extramammary Paget's disease (EMPD) is a rare, intraepithelial adenocarcinoma that primarily affects the anogenital and axillary regions. Wide local excision is the standard first-line treatment. However, due to the frequently ill-defined margins and subclinical extension of the disease, positive surgical margins and high local recurrence rates (ranging from 4.8% to 57.6%) are common challenge (1). In cases where re-excision is not feasible without compromising critical anatomical structures or function, effective non-surgical alternatives are urgently needed.

Topical PDT works for some superficial EMPD cases, but not consistently. This inconsistency is largely due to the poor cutaneous penetration of topically applied photosensitizers, which fail to reach EMPD cells residing deep within adnexal structures such as hair follicles and glands (2). Its efficacy is often limited by the poor penetration of the photosensitizer through the stratum corneum and into deeper adnexal structures, which are frequently involved in EMPD. To overcome this barrier, intralesional PDT (iPDT) has been proposed as a method to deliver the photosensitizer directly into the lesion, ensuring higher and more homogeneous drug distribution at the target site (3). We herein report a successful case of residual EMPD with positive margins after surgery that achieved complete clinical regression following intralesional aminolevulinic acid (ALA)-PDT.

## Case presentation

**Figure 1:**
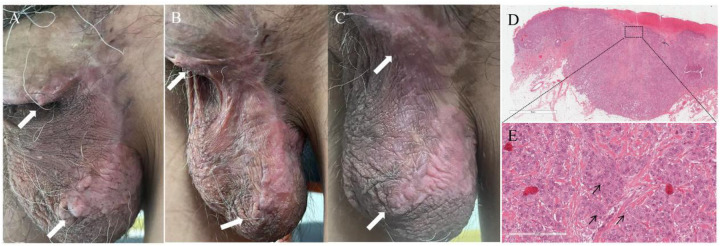
Clinical and histopathological features before and after iPDT. **(A)** Clinical presentation before treatment showing an infiltrated erythematous plaque surrounding the surgical scar. **(B)** Clinical appearance after 3 sessions of iPDT, demonstrating marked thinning and fading of the lesion. **(C)** Clinical appearance after 6 sessions of iPDT, showing complete regression with residual post-inflammatory hypopigmentation (white arrows). **(D,E)** Histopathology (H&E staining) before treatment: **(D)** Low magnification (scale bar = 3 mm) showing dermal infiltration of Paget cells; **(E)** High magnification (× 400, scale bar = 200 μm) revealing atypical Paget cells with abundant pale cytoplasm and pleomorphic nuclei (black arrows).

A male patient in his 60 s presented with a history of extramammary Paget's disease of the scrotum. He underwent surgical excision of the lesion in the urology department. The initial surgery was a standard wide local excision performed by the urology department under local anesthesia. The surgical margins were drawn approximately 1 cm beyond the visible border of the lesion. The excision was carried down to the subcutaneous fat, and the defect was primarily closed. Postoperatively, the wound healed with a scar, but thickened, infiltrative erythematous patches with indistinct borders persisted around the scar, indicative of residual disease. Histopathological examination of the excised specimen confirmed the diagnosis of EMPD, with tumor cells infiltrating the subcutaneous fat tissue. Figure 1A (Clinical, pre-iPDT) showed the persistent, thickened, erythematous plaque surrounding the surgical scar. Histopathological examination (Figures 1D,E) confirmed the infiltration of the dermis and subcutaneous fat by atypical Paget cells with abundant pale cytoplasm and pleomorphic nuclei, consistent with residual disease.

**Figure 2:**
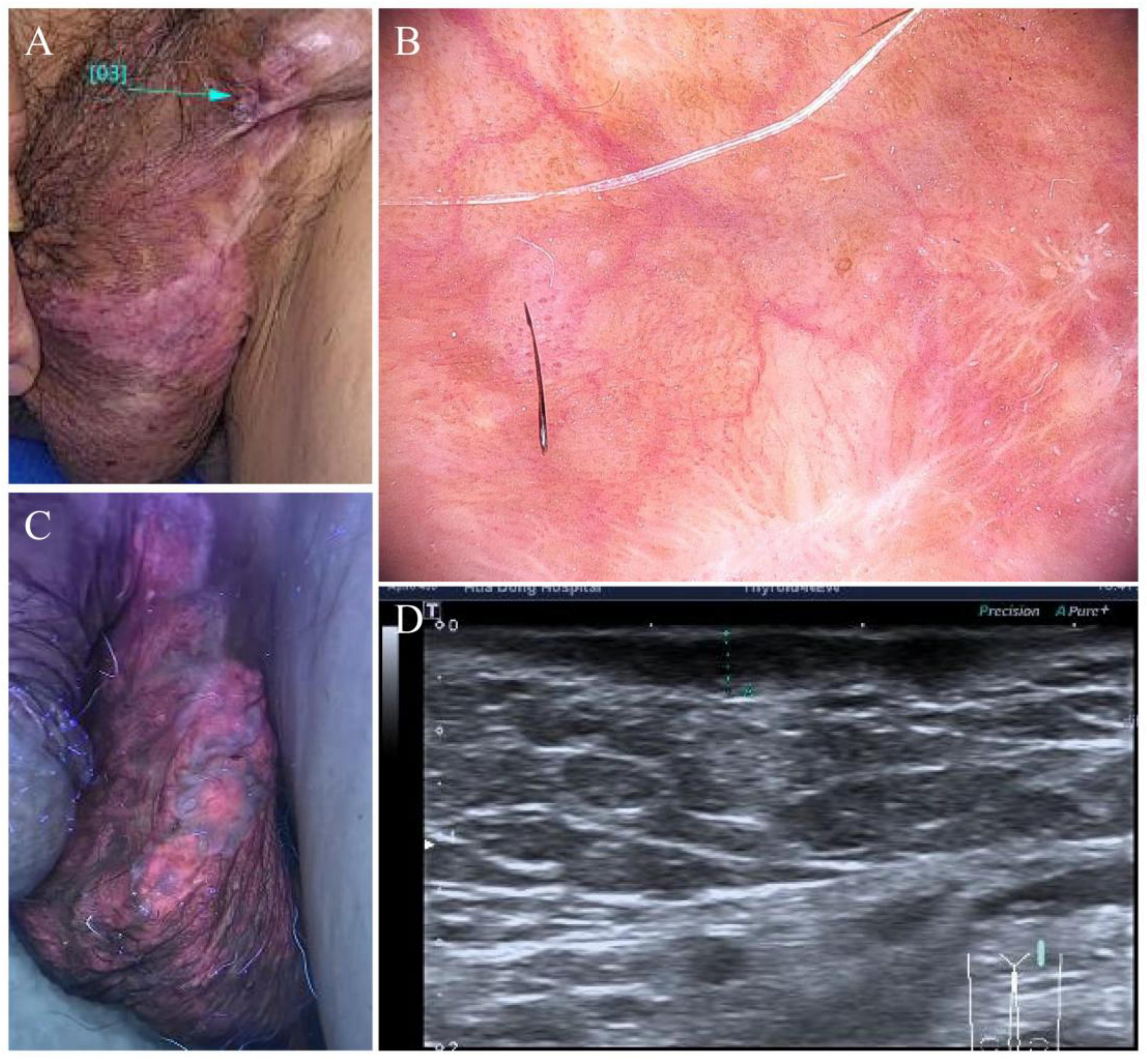
Multimodal imaging of residual EMPD before treatment. **(A)** Clinical image of the scrotal lesion; the green box indicates the area examined by dermoscopy. **(B)** Dermoscopy showing a polymorphous vascular pattern (including glomerular vessels) and milky-red areas against a yellowish background. **(C)** Intense red fluorescence under Wood's lamp examination 2 hours after intralesional ALA injection, confirming homogeneous and deep PpIX accumulation within the lesion, thereby validating successful drug delivery to the full depth of the tumor. **(D)** High-frequency ultrasound (B-mode, 20 MHz) revealing a heterogeneous, hypoechoic lesion extending into the mid-dermis, with a thickness of approximately 2.7 mm. All images have been anonymized and no unique patient identifiers are visible.

Critically, both the lateral and deep (subcutaneous) margins were positive for Paget cells, indicating incomplete resection. Pre-treatment monitoring with dermoscopy (Figure 2B) revealed a polymorphous vascular pattern with milky-red areas, and high-frequency ultrasound (Figure 2D) demonstrated a heterogeneous, hypoechoic lesion extending into the mid-dermis, corroborating deep involvement. However, further wide local excision was deemed impractical due to the high risk of significant functional and structural damage to the genitalia. Given this dilemma, the patient was referred to the dermatology department for local adjuvant therapy. After comprehensive discussion and obtaining informed consent, a regimen of intralesional ALA-PDT was initiated.

A 10% (100 mg/mL) solution of 5-aminolevulinic acid (ALA) was prepared by dissolving ALA powder in 0.9% sterile saline immediately before use. This concentration was selected based on a 2025 systematic review identifying 10% ALA-PDT as an effective regimen for basal cell carcinoma and other cutaneous malignancies (4), as well as a phase III trial demonstrating a 75.9% histological clearance rate for superficial basal cell carcinoma using 10% ALA gel (5). Under aseptic conditions, the solution was injected intralesionally into the entire residual erythematous and indurated plaque using a 30-gauge needle. The injection was performed until a slight blanching and wheal formation were observed. Approximately 0.1–0.2 mL of the ALA solution was injected per puncture point, with adjacent injection points spaced about 5–8 mm apart. The area was then covered with an opaque dressing to protect it from light for an incubation period of 2 hours. Because intralesional injection bypasses the stratum corneum, the photosensitizer reaches the target cells almost immediately, and a 2-hour incubation is sufficient for PpIX accumulation, as confirmed by the intense fluorescence observed. After incubation, intense red fluorescence was observed under Wood's lamp examination (Figure 2C), indicating robust protoporphyrin IX (PpIX) accumulation within the tumor. The lesion was then illuminated using a 630 nm red light-emitting diode (LED) device. A light dose of 120 J/cm^2^ was delivered at an irradiance of 66.7 mW/cm^2^, corresponding to an irradiation time of 30 minutes per session. This procedure was repeated once per month, a schedule chosen to balance treatment efficacy with adequate recovery time based on patient preference and previous iPDT protocols (6). Clinical assessment was performed before each session. The patient tolerated the procedures well. The most common side effect was mild to moderate pain during irradiation, which was managed with cold air analgesia. Transient erythema and edema in the treated area resolved within a week after each session.

After three sessions of iPDT, a significant clinical improvement was noted. The infiltrative plaque showed marked thinning, and the erythema had noticeably faded (Figure 1B). After six sessions, the lesion showed complete clinical regression, leaving behind only flat, supple skin with mild post-inflammatory hypopigmentation (Figure 1C). No recurrence was observed during the subsequent 6-month follow-up period.

### Ethics statement

This case report describes the routine clinical management of a single patient. The treatment was performed as part of standard medical care, not as a research study. Written informed consent was obtained from the patient for the treatment and for the publication of this report, including all clinical images. According to Article 38 of the Chinese “Measures for Ethical Review of Biomedical Research Involving Human Subjects” (2016), retrospective case reports derived from routine clinical practice that involve no more than the analysis of a single patient's de-identified data may be exempt from formal ethical review. This report was prepared in accordance with the principles of the Declaration of Helsinki. A copy of the patient's written informed consent is available upon request.

## Discussion

The management of residual EMPD presenting with deep dermal invasion presents a distinct therapeutic challenge. While wide local excision remains the gold standard, the positive surgical margins in this case—with tumor cells infiltrating the subcutaneous fat—rendered re-excision impractical due to the high risk of functional compromise to the genitalia. This clinical dilemma prompted the exploration of non-surgical, tissue-sparing alternatives.

Conventional topical PDT, though effective for superficial cutaneous neoplasms, often fails in EMPD due to its limited penetration depth (typically 1–2 mm) (3). In the present case, high-frequency ultrasound (20 MHz) objectively quantified the lesion thickness at approximately 2.7 mm, with a heterogeneous hypoechoic pattern extending into the mid-dermis (Figure 2D). This finding objectively confirmed that the tumor burden exceeded the therapeutic reach of topical photosensitizer application. Furthermore, dermoscopy revealed a polymorphous vascular pattern with milky-red areas and glomerular vessels (Figure 2B)—features previously correlated with invasive or deeply infiltrative EMPD (7). These imaging modalities collectively provided a strong rationale for an intralesional approach. Given the deep infiltration and the fact that re-excision was deemed unfeasible due to high risk of functional/structural damage to the genitalia, we considered the main non-surgical alternatives: imiquimod cream (mainly effective for superficial lesions and requiring prolonged application with local irritation), radiotherapy (effective but carries risks of long-term irradiation dermatitis and potential urethral stricture), and topical PDT. Among these, we selected intralesional ALA-PDT because (i) the 2.7 mm thickness exceeded the penetration depth of all topical agents; (ii) direct intralesional injection ensures homogeneous photosensitizer delivery to the full tumor depth, overcoming the key limitation of topical PDT; and (iii) it is minimally invasive, repeatable, and tissue-sparing.

Intralesional ALA-PDT directly addresses this penetration barrier. By injecting the photosensitizer throughout the entire indurated plaque, high and homogeneous concentrations of ALA are achieved at the full depth of the tumor. The subsequent intense, uniform red fluorescence observed under Wood's lamp (Figure 2C) served as a functional confirmation of successful PpIX accumulation within the target tissue, including its deepest components. This stands in clear contrast to topical PDT, where fluorescence is often patchy and confined to the superficial layers (2).

The clinical outcome—complete regression after six sessions—underscores the potency of this approach. Notably, the response was gradual rather than immediate: significant thinning was observed after three sessions, with complete regression only after six. This temporal pattern suggests that beyond direct photocytotoxicity, repetitive iPDT sessions may have harnessed an anti-tumor immune response (8, 9). PDT-induced immunogenic cell death can release tumor-associated antigens and damage-associated molecular patterns (DAMPs), potentially activating host immune surveillance against residual microscopic disease—a particularly valuable mechanism for EMPD, which is known for its field cancerization and irregular subclinical extension (10, 11).

The rationale for intralesional delivery is further supported by its successful application in other dermatological conditions characterized by deep-seated pathology. For instance, iPDT has shown promising results in treating recalcitrant deep fungal infection (12) and pilonidal sinus disease (13), where topical agents similarly fail to penetrate to the site of active disease. These conditions share a common challenge with EMPD: pathology extending beyond the reach of surface-applied therapy, thereby reinforcing the rationale for direct intralesional administration. This cross-disease evidence suggests that the principle of bypassing penetration barriers via injection is broadly applicable to deep cutaneous lesions, further validating its use in the present case of infiltrative EMPD. The homogeneous drug distribution achieved via injection minimizes the risk of subtherapeutic “pockets”, which is critical for a disease like EMPD known for its irregular subclinical extension (6). The mechanism of iPDT likely involves not only direct cytotoxic effects on tumor cells and damage to the tumor's microvasculature, but also the induction of an anti-tumor immune response, as suggested by the gradual, cumulative clinical improvement observed over multiple sessions (8, 9).

We acknowledge that no post-operative pre-PDT biopsy nor post-treatment histologic confirmation was obtained, due to patient preference and the sensitive anatomic location. Furthermore, post-treatment ultrasound was not performed because the equipment was unavailable at follow-up visits; therefore objective volumetric data to confirm the reduction of lesion thickness are lacking. Consequently, we use the term “clinical remission” rather than “cure”. In addition, the follow-up period is limited to six months, which is insufficient for EMPD given its potential for late recurrence. The patient remains under ongoing surveillance with regular clinical examinations (every 3–6 months) at our department, and we plan to follow the patient for at least 36 months. Future studies should incorporate both pre-treatment confirmatory biopsy and post-treatment objective assessment (histology, ultrasound, or reflectance confocal microscopy) when feasible.

## Conclusion

This case provides compelling evidence that intralesional ALA-PDT is a highly effective and tissue-preserving therapeutic modality for the management of residual or recurrent EMPD, especially in anatomically constrained surgical sites where further excision is not viable. It successfully overcomes the key limitations of topical PDT by ensuring adequate drug delivery to the entire tumor burden. We propose that iPDT should be considered a valuable tool in the multidisciplinary management of this challenging disease. Given its efficacy and tissue-sparing nature, iPDT deserves consideration as a first-line adjuvant option for selected patients with residual EMPD. While histologic confirmation was not obtained, the sustained clinical remission at 6 months suggests that iPDT is highly effective. Further larger-scale studies are warranted to standardize the treatment protocol and validate its long-term efficacy.

## Data Availability

The raw data supporting the conclusions of this article will be made available by the authors, without undue reservation.
